# New-onset gastrointestinal disorders in COVID-19 patients 3.5 years post-infection in the inner-city population in the Bronx

**DOI:** 10.1038/s41598-024-83232-7

**Published:** 2024-12-30

**Authors:** Sagar Changela, Samad Ashraf, Justin Y. Lu, Kevin E. Duong, Sonya Henry, Stephen H. Wang, Tim Q. Duong

**Affiliations:** 1 Department of Radiology, Albert Einstein College of Medicine and Montefiore Medical Center, 1300 Morris Park Avenue, Bronx, NY 10461 USA; 2 Department of Surgery, Beth Israel Deaconess Medical Center, Harvard Medical School, Boston, MA USA; 3 Center for Health & Data Innovation, Albert Einstein College of Medicine and Montefiore Medical Center, Bronx, NY USA

**Keywords:** long covid, post-acute sequelae of COVID-19 (PASC), health disparity, SARS-CoV-2, Epidemiology, Gastrointestinal diseases, Viral infection

## Abstract

This study examined the incidence, characteristics, and risk factors of new gastrointestinal disorders (GID) associated with SARS-CoV-2 infection up to 3.5 years post-infection. This retrospective study included 35,102 COVID-19 patients and 682,594 contemporary non-COVID-19 patients without past medical history of GID (controls) from the Montefiore Health System in the Bronx (3/1/2020 to 7/31/2023). Comparisons were made with unmatched and propensity-matched (1:2) controls. The primary outcome was new GID which included peptic ulcer, inflammatory bowel disease, irritable bowel syndrome, diverticulosis, diverticulitis, and biliary disease. Multivariate Cox proportional hazards model analysis was performed with adjustment for covariates. There were 2,228 (6.34%) COVID-19 positive patients who developed new GID compared to 38,928 (5.70%) controls. COVID-19 patients had an elevated risk of developing new GID (adjusted HR = 1.18 (95% CI 1.12–1.25) compared to propensity-matched controls, after adjusting for confounders that included smoking, obesity, diabetes, hypertension. These findings underscore the need for additional research and follow-up of at-risk individuals for developing GID post infection.

**Supplementary Information:**

The online version contains supplementary material available at 10.1038/s41598-024-83232-7.

## Introduction

SARS-CoV-2 infection, although primarily affects the respiratory system^1–3^, is associated with a range of acute gastrointestinal (GI) symptoms^4–7^. These acute GI symptoms include diarrhea, nausea, vomiting, abdominal pain, and loss of appetite, and are reported in a significant number of COVID-19 patients^4–7^. There is evidence that SARS-CoV-2 virus enters epithelial cells in the GI tract via the ACE2 receptor and replicate in the GI tract^8–10^. This is not surprising as the influenza virus (a coronavirus) also has GI manifestations and is known to replicate in human intestinal tissue^11–13^. Viral ribonucleic acid (RNA) has also been detected in the stools of patients with confirmed influenza^14,15^.

Although GI symptoms have been reported during acute SARS-CoV-2 infection^4–7^, whether these symptoms persist post COVID-19 and whether SARS-CoV-2 infection results in increased incidence of new GI disorders (GID) post infection is mostly unknown. These GID could include inflammatory bowel disease (IBD), irritable bowel syndrome (IBS), diverticulitis, gastritis, pancreatitis, peptic ulcer, Crohn’s disease, ulcerative colitis etc^5,16–19^. The relative risk of SARS-CoV-2 infection compared to known risks of GID are unknown. Known risks include demographic factors (i.e., old age, female sex, race, ethnicity) and pre-existing conditions (smoking, diabetes, hypertension, chronic kidney disease (CKD), asthma, and obesity). It is unclear whether SARS-CoV-2 infection exerts differential effects on GID post infection in different racial or ethnic subgroups as well as individuals with pre-existing medical conditions. Exploring these associations could provide critical insights into the longer-term GI effects of COVID-19.

The goal of this study was to investigate the incidence of new-onset GID in patients post COVID-19 compared to matched controls without COVID-19 up to 3.5 years post COVID-19. We also identified risk factors for developing new-onset GID. Data came from the Montefiore Health System in the Bronx and its environs which serves a diverse population, and which was one of the epicenters of early COVID-19 pandemic and its subsequent waves.

## Method

This retrospective cohort study with waived informed consent was approved by the Institutional Review Board (2021–13658) of the Albert Einstein College of Medicine-Montefiore Health System in accordance with all relevant guidelines and regulations of the Albert Einstein College of Medicine. Informed consent was waived for this study by the Institutional Review Board (2021–13658) of the Albert Einstein College of Medicine. Data (March 2020 to July 2023) came from the Montefiore Health System which consisted of several hospitals and outpatient clinics in the Bronx and its environs that a diverse population. Data were extracted as described previously^20,21^. To ensure data accuracy, we performed extensive cross-validation of all major variables by manual chart review of subsets of patients. Studies using an earlier version of this large database to address different questions have been published^20–39^.

COVID-19 patients were those with a positive polymerase-chain-reaction (PCR) test for COVID-19. Controls were patients without any history of a positive COVID-19 test or documentation of COVID-19 in our health system. The index date was defined as the date of the PCR test for the COVID-19 cohort or the first healthcare encounter after March 1, 2020, for the control cohort. Patients who did not return to our health system 14 days post index date or later were excluded. Propensity score matching (1:2 experimental: control group) was performed for patients who returned to the health system at least 14 days after the index date, using age (within ± 5 years), sex, race, and ethnicity.

Demographic data, such as age, sex, race, and ethnicity, as well as pre-existing comorbidities (diabetes, hypertension, COPD, CKD, cardiovascular disease, asthma, and obesity) were extracted. The independent variable was COVID-19 status. The dependent variable was GI conditions, which included esophagitis, gastritis, peptic ulcer, ulcerative colitis, irritable bowel syndrome, irritable bowel disease, diverticulosis, diverticulitis biliary disease, etc. (Supplemental Table 1).

The primary outcome was new-onset GI conditions. Sociodemographic factors were compared between COVID-19 and controls. Cumulative incidence curves for outcomes of the two groups were constructed with accounts for competing risk (death). Univariate and multivariate Cox-proportional hazard analyses for new onset GI conditions were performed for unmatched and matched data.

### Statistics

All the statistical analysis was performed on SAS system version 9.4 (SAS Institute Inc., Cary, NC) and R version 4.3.2. Analysis of group differences employed chi-square tests for categorical variables and unpaired t-tests for continuous variables. When comparing differences of categorical variables across three groups, chi-square tests were used to identify group differences and ad hoc pairwise tests were used to identify the specific pairwise group difference. Statistics were not adjusted for multiple comparison due to the exploratory nature of the study. All statistical tests were two-sided, and *P* values less than 0.05 were considered statistically significant.

## Results

**Fig. 1:**
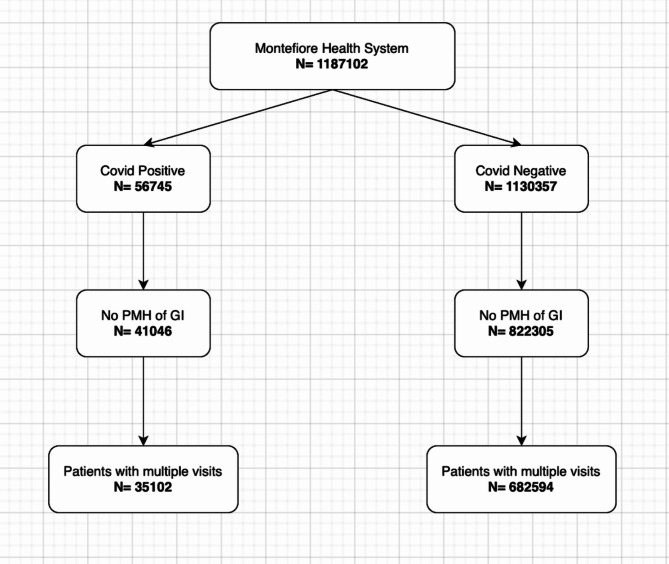
Flowchart of cohort selection. After determining which patients were positive for COVID-19 versus COVID-19 negative, patients were excluded based on prior gastrointestinal history. Additionally, COVID-19 negative patients were identified after the index date determined in our study as March 2020.

Figure 1 shows the patient selection flowchart. The study consisted of 1,187,102 individuals from Montefiore health system, of which 56,745 were COVID-19 positive and 1,130,357 were COVID-19 negative. After excluding individuals with prior GID, there were 41,046 COVID-19 positive patients and 822,305 COVID-19 negative patients, of which 35,102 COVID-19 positive patients and 682,594 COVID-19 negative patients return to our health system post index date.

Table 1 summarizes the distribution of new-onset GID cases, demographics and comorbidities of COVID-19 positive and negative cohort (not matched data). There were 2,228 (6.34%) COVID-19 positive and 38,928 (5.7%) COVID-19 negative who had new GI. COVID-19 positive patients were older (41.79 vs. 39.76, *p* < 0.001) more likely active smokers (14.51% vs. 13.13%, *p* < 0.001) and had high prevalence of all major comorbidities (*p* < 0.001) compared to COVID-19 negative patients. Both the cohort has higher proportions of female (56.18% and 55.77%, respectively) and non-Hispanic patients (59.05% and 65.11%, respectively).

**Table 1:** Profiles of all patients with and without COVID-19 (unmatched cohorts).

	Covid positive *N* = 35,102	Covid negative *N* = 682,594	Chi-square
New GI Conditions	2228 (6.34)	38,928 (5.70)	< 0.001
Acid related	1619 (4.61)	28,253 (4.14)	
IBD/IBS	74 (0.21)	1839 (0.27)	
Diverticulosis and diverticulitis	442 (1.26)	7775 (1.14)	
Biliary disease	355 (1.01)	5575 (0.81)	
**Age (Mean)**	41.79	39.76	< 0.001
Gender			< 0.001
Male	15,383 (43.82)	301,679 (44.19)	
Female	19,722 (56.18)	380,685 (55.77)	
Race			< 0.001
White	3781 (10.77)	86,630 (12.69)	
Black	11,168 (31.81)	184,077 (26.97)	
Asian	1374 (3.91)	16,998 (2.49)	
Others	212 (0.6)	3951 (0.58)	
Unknown	18,570 (52.89)	390,938 (57.27)	
Ethnicity			< 0.001
Hispanic	14,376 (40.95)	237,964 (34.86)	
Non- hispanic	20,729 (59.05)	444,429 (65.11)	
Smoking			< 0.001
Never/Missing	27,489 (78.31)	549,308 (80.47)	
Former	2524 (7.19)	43,620 (6.39)	
Yes	5092 (14.51)	89,666 (13.13)	
Comorbidities			
Diabetes	8553 (24.37)	88,517 (12.97)	< 0.001
Hypertension	11,231 (31.99)	130,179 (19.07)	< 0.001
CKD	3310 (9.43)	24,190 (3.54)	< 0.001
COPD	1208 (3.44)	7592 (1.11)	< 0.001
Cardiovascular diseases	3890 (11.08)	32,989 (4.83)	< 0.001
Asthma	5963 (16.98)	68,110 (9.98)	< 0.001
Obesity	6839 (19.48)	77,422 (11.34)	< 0.001

Frequencies and percentages for categorical variables between cohorts were compared using chi-squared tests. *p* < 0.001.

**Fig. 2:**
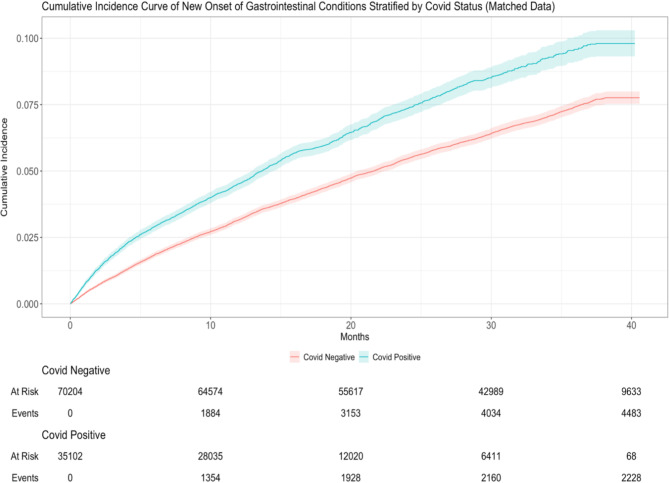
Cumulative incidence curve of new onset of gastrointestinal conditions stratified by patients with COVID-19 to non-COVID-19 patients (matched cohort). Cohort data were matched by age, sex and ethnicity.

COVID-19 negative patients were matched with COVID-19 positive patients by age, sex, race and ethnicity (1:2 match) using propensity score matching. Figure 2 represents the cumulative incidence curve of developing new GI disorders stratified by COVID-19 status with accounting for competing risk (death) for the matched cohorts. COVID-19 cohort showed higher new incident GI compared to the non-COVID-19 cohort.

Table 2 shows the results of Cox proportional hazard model for matched data. Crude model illustrates that hazard of having new GI was 1.37 (95% CI 1.30–1.44) time higher among COVID-19 positive compared to COVID-19 negative patients. Smoking, diabetes, hypertension, CKD, asthma and obesity were significantly associated with developing new GID in the multivariate adjusted model. After accounting for these covariates, the adjusted HR for new GID for COVID-19 status was 1.18 (95% CI 1.12–1.25). Results of the Cox-proportional hazard model of the unmatched data, shown in Supplementary Table 2, were similar to those of the matched data.

**Table 2:** Crude and multivariable Cox-proportional hazard ratios of matched cohorts.

Variable	Crude Hazard ratio	95% CI	Adjusted Hazard ratio	95% CI
Covid positive vs. Covid negative	1.37*	(1.30–1.44)	1.18*	(1.12–1.25)
Smoker vs. Never	1.87*	(1.78–1.96)	1.50*	(1.42–1.58)
Diabetes	2.14*	(2.04–2.26)	1.36*	(1.27–1.44)
Hypertension	2.24*	(2.13–2.35)	1.59*	(1.49–1.69)
COPD	2.14*	(1.89–2.42)	1.02	(0.89–1.17)
CKD	2.10*	(1.95–2.26)	1.13*	(1.04–1.23)
Cardiovascular diseases	1.94*	(1.80–2.08)	1.06	(0.97–1.15)
Asthma	1.30*	(1.21–1.38)	1.13*	(11.06–1.21)
Obesity	1.77*	(1.67–1.87)	1.28*	(1.20–1.36)

Cohort data were matched by age, sex and ethnicity.

To investigate the potential effects of different viral strains on outcomes, incidences of new GID for different predominant strains with one-year observation post infection were tabulated (Supplemental Table 3). The highest GID incidence was observed in the Alpha strain period (3.91%), while the lowest was during the Delta strain period (2.48%). Incidences of new GID among infections by the original, Alpha, Delta and Omicron strain were similar, except that of Delta was lower than those of others (*p* < 0.05, Chi square).

## Discussion

This study investigated the incidence of new GID and identified risk factors contributing to the development of these disorders up to 3.5 years post-infection. We found that 6.34% (2,228) of COVID-19-positive patients developed new GI disorders, compared to 5.70% (38,928) of controls, with COVID-19 patients being more likely to develop new GI disorders than matched controls (adjusted HR = 1.18, 95% CI [1.12–1.25]).

### Acute GI manifestations

Many studies have documented GI symptoms during acute COVID-19. A systematic review of 16 studies including a total of 3646 patients found that the most commonly presenting acute GI symptom in patients with COVID-19 were diarrhea (47% of patients), followed by anorexia (19%)^4^. Other common acute symptoms included nausea, vomiting and abdominal pain. Another systematic review by Kariyawasam et al. reported that the most common acute GI symptoms were anorexia, diarrhea, nausea, vomiting and abdominal pain/discomfort^5^. These acute GI manifestations were reported in 11.4–61.1% of individuals with COVID-19. A study conducted by Han et al., 2020 investigated the digestive symptoms within 206 patients with low-severity COVID-19 and found that 67 patients presented with diarrhea which lasted from 1 to 14 days^7^. This study also tested stool RNA within 22 COVID-19 infected patients and determined that 12 (54.5%) patients tested positive for viral RNA in stool.

### Post-acute GI manifestations

By contrast, studies of persistent GI symptoms and new incident GID post infection are sparse. Cheung et al. found 10–20% of COVID-19 patients experienced persistent GI symptoms after the resolution of respiratory manifestations^40^. Austhof et al. found that of the 1475 participants in the Arizona Cohort, 33.8% (*n* = 499) had acute GI symptoms. The odds of developing persisting GI symptoms > 45 days post infection in patients with acute GI symptoms was 4 times higher than those without acute GI symptoms (OR = 4.29. 05% CI 2.45–7.53)^16^. Hawkings et al. performed a systematic review of literature from Dec 2019 to Jul 2023 and reported a prevalence for persistent GI symptoms of 10.8% in COVID-19 patients compared with 4.9% in healthy controls^41^. Golla et al. compared presence of GI symptoms in a 6-month follow-up period in 320 patients with COVID-19 to 320 matched healthy spouses/family member controls and 280 healthy COVID-19 negative controls^19^. Of the 320 COVID-19 cases, 36 (11.3%) developed GI at 1 month follow-up with persistent symptoms in 27 (9.4%) patients at 3 months and 21 (6.6%) patients at 6 months. Notably, none of the healthy controls (320 healthy spouses/family members and 280 healthy COVID-19 negative controls) had GI symptoms up to 6 months of follow-up (*p* < 0.01). Karime et al. reported that patients with diverticulitis at the time of COVID-19 diagnosis had higher complication rates of intestinal perforation (55.9% vs. 27.7%, *p* = 0.01), peritonitis (29.4% vs. 4.3%, *p* < 0.01), and the need for emergent surgical intervention (38.2% vs. 10.6%, *p* < 0.01) within 30 days of COVID-19 diagnosis (*N* = 47) between 2020 and 2022 from Mayo Clinic^18^. There are only a few reports on new GID post infection. One such report is by Xu et al. who studied the VA population of 154,068 people with COVID-19, 5,638,795 contemporary controls, and 5,859,621 historical controls, and found that people with COVID-19 showed increased risks of GID (dyspepsia, gastroesophageal reflux disease, peptic ulcer disease, functional intestinal disorders, acute pancreatitis, hepatic and biliary disease), with a hazard ratio of any new incidence of 1.37 (95% CI 1.33,1.41) 1-year post infection^17^.

Although patients infected by the original SARS-CoV-2 strain were more likely to have acute severe COVID-19 outcomes compared to other strains^42^, the original strain resulted in a slightly, but not significantly, higher incidence of new GID post infection compared to other predominant strains. Only the incidence of new GID of the Delta was significantly lower than those of other strains.

### Possible mechanisms of post-infection GID

Post-infection GID could be a result of direct viral invasion, hyperinflammation, microbiome dysbiosis, side effects of COVID-19 treatment, and psychological stress. There is evidence of direct SARS-CoV-2 infection in the GI system via the angiotensin-converting enzyme 2 (ACE2) receptors in the GI system^43–46^. Zou et al. used single-cell RNA sequencing to identify organs vulnerable to SARS-CoV-2, finding that esophageal and ileal epithelial cells, which have high ACE2 levels, are at high risk^9^. Qi et al. found high ACE2 expression in liver cholangiocytes, colon colonocytes, esophagus keratinocytes, ileum and rectum endothelial cells, and stomach epithelial cells^47^.

COVID-19 can trigger hyperinflammation, including in the GI tract^48^. This response can damage the gut lining, leading to increased intestinal permeability or “leaky gut.” This condition allows harmful substances to enter the bloodstream, causing chronic inflammation and potentially leading to new GI disorders such as irritable bowel syndrome (IBS) and functional dyspepsia^49^.

The gut microbiome, essential for GI health, can be significantly disrupted by COVID-19 and its treatment. This disruption can lead to an imbalance between beneficial and harmful bacteria, known as dysbiosis. Dysbiosis can contribute to GI disorders like inflammatory bowel disease (IBD) and small intestinal bacterial overgrowth (SIBO) by promoting inflammation and impairing gut function^50^.

The treatment of COVID-19 could have adverse effects on GI health. Antiviral medications, antibiotics, corticosteroids, immunomodulators, and supportive care drugs can all contribute to GI symptoms and disorders^51,52^.

The psychological impact of COVID-19, including stress, anxiety, and depression, can also indirectly affect GI health. The gut-brain axis, a communication network between the gut and the brain, can be disrupted by psychological stress, leading to or exacerbating functional GI disorders^53,54^. Chronic stress can alter gut motility, increase gut sensitivity, and affect the gut microbiome’s composition^53,54^.

### Other covariates

We found that smoking status, diabetes, hypertension, CKD, asthma and obesity to be significant covariates contributing to outcomes. Most of these are known risk factors for GID. For examples, smoking is linked to GI cancers, peptic ulcers, and inflammatory bowel disease, while diabetes is associated with gastroparesis, non-alcoholic fatty liver disease, and higher risks of liver and pancreatic cancers^55–59^. Hypertension can exacerbate GID like gastroesophageal reflux disease (GERD), especially when treated with medications that increase ulcer risk^60^. At the same time, CKD is linked to gastritis and GI bleeding due to altered medication metabolism^61^. Obesity is a major risk factor for GERD, gallstones, and colorectal cancers, and asthma is commonly associated with GERD^62^.

Our analysis of unmatched data revealed that demographic factors significantly influenced new GID outcomes. Older age was associated with a higher risk for GID, and females had an elevated risk, consistent with previous findings. Black and Asian individuals also showed a higher risk of new GID compared to White individuals, as did Hispanic individuals compared to non-Hispanic individuals. COVID-19 has disproportionately impacted marginalized communities^63,64^, likely due to limited healthcare access, higher prevalence of underlying health conditions, crowded living conditions, and employment in essential but high-risk roles have compounded the burden of COVID-19 within already vulnerable populations. Our findings suggest that COVID-19 imposed additional disease burden on these groups. Investigating the relationship between demographic factors and post-infection GID may illuminate specific challenges faced by these populations and inform strategies to meet the complex needs of those experiencing lasting health effects following SARS-CoV-2 infection^65^.

### Other respiratory viruses

Acute and post-acute GI manifestations have been reported to be associated with other respiratory virus infections. For example, the influenza virus has been previously found to have been detected in stool samples^14,15^. Minodier et al. studied the GI effects of influenza, coronavirus, rhinovirus, RSV, adenovirus in 331 patients and found that among the 104 patients with influenza, 56.7% presented with GI symptoms^12^. Notably, 78.9% of patients with human coronavirus infection had GI infections. A systematic review also conducted by Minodier et al. analyzed 44 studies looking at the pooled prevalence of acute digestive symptoms such as diarrhea, nausea, vomiting, abdominal pain in addition to more severe gastrointestinal effects such as acute appendicitis and hemorrhagic gastritis^11^. This value ranged from 30.9% (95% CI, 9.8 to 57.5; I^2^ = 97.5%) for A(H1N1) pdm09 to 2.8% (95% CI, 0.6 to 6.5; I^2^ = 75.4%) for A(H1N1). In an animal study, IFN-γ produced by lung-derived CCR9 + CD4 + T cells associated with influenza viral infection are recruited into the small intestine, which disrupted the intestinal microbiota^66^.

### Limitations

The strengths and novelties of our study included analysis of new GID (as opposed to symptoms), longer-term post infection follow-up (up to 3.5 years), large sample size, and a diverse patient cohort. We also employed analysis of controls with propensity matching. There are several limitations in our analysis. Our findings were limited to patients who returned to our health system. It is possible that patients who returned were more likely to have more severe COVID-19. However, patient records included those who returned for any medical reason, including but not limited to routine office visits. Electron medical records could have errors including missing data and misclassifications of COVID-19 positive versus COVID-19 negative case. We did not analyze various treatments with respective to outcomes. This was because each patient could have undergone many treatments that could include oxygen therapy, corticosteroids, intubation, invasive mechanical ventilation, antiviral medications, and monoclonal antibody medications, among others. Moreover, these treatments were administered in different combinations and might not have been consistently applied in early pandemic in a control manner to allow such analysis. Vaccine status, which could affect outcomes, was not reliably recorded if patients received vaccine outside our healthcare system. Thus, vaccine status was not incorporated into our model. As with any retrospective study, there could be other unintended patient selection biases and latent confounding factors.

## Conclusions

COVID-19 patients are more likely to develop new GI disorders compared to contemporary matched controls. These findings underscore the need for ongoing research and follow-up care strategies for post SARS-CoV-2 to improve GI health.

## Electronic Supplementary Material

Below is the link to the electronic supplementary material.

Supplementary Material 1.

Supplementary Material 2.

Supplementary Material 3.

## Data Availability

Data can be provided upon reasonable request.
