# Supplementary material for: New-onset gastrointestinal disorders in COVID-19 patients 3.5 years post-infection in the inner-city population in the Bronx
Source: Sci Rep. 2024 Dec 30;14:31850. doi: 10.1038/s41598-024-83232-7 (PMC11685902; doi:10.1038/s41598-024-83232-7)
Supplement: Supplementary file 1 — Supplementary Material 1. [file 41598_2024_83232_MOESM1_ESM.pdf]

Supplementary Table 1: Pre-set gastrointestinal-related search terms utilized to create the cohort in this retrospective analysis.

CONCEPT_ID	CONCEPT_NAME
27918	Eosinophilic esophagitis
30437	Gastro-esophageal reflux disease with esophagitis
30753	Esophagitis
75576	Irritable bowel syndrome
75580	Chronic ulcerative proctitis
77317	Chronic ulcerative rectosigmoiditis
81893	Ulcerative colitis
192363	Obstruction of gallbladder
192667	Atrophic gastritis
192956	Cholecystitis
193239	Diverticulitis of small intestine
193249	Acute hemorrhagic gastritis
193250	Gastric hemorrhage
193252	Diverticulosis of small intestine
194684	Crohn's disease of large bowel
195306	Gastroduodenitis
195307	Hydrops of gallbladder
195575	Crohn's disease of small AND large intestines
195585	Crohn's disease of small intestine
195587	Cholelithiasis AND cholecystitis with obstruction
195856	Cholangitis
196456	Gallstone
196469	Perforation of gallbladder
196726	Gallbladder calculus with acute cholecystitis and no obstruction
197924	Bile duct calculus with acute cholecystitis and obstruction
198809	Acute cholecystitis
199866	Acute gastritis
200154	Fistula of gallbladder
200444	Postcholecystectomy syndrome
200765	Chronic cholecystitis
201340	Gastritis
201352	Gallbladder calculus with acute cholecystitis and obstruction
201606	Crohn's disease
201614	Calculus of bile duct with cholecystitis

318186	Achalasia of esophagus
433516	Duodenitis
442750	Calculus of bile duct with acute cholecystitis without obstruction
443421	Gallbladder and bile duct calculi
443785	Calculus of gallbladder with cholecystitis
443889	Calculus of bile duct with chronic cholecystitis with obstruction
444122	Calculus of bile duct with obstruction
444367	Cholelithiasis without obstruction
4009168	Gangrene of gallbladder
4027663	Peptic ulcer
4027729	Acute duodenal ulcer with hemorrhage
4046500	Acute peptic ulcer with hemorrhage
4055341	Calculus of bile duct with cholangitis
4057513	Chronic superficial gastritis
4057826	Irritable bowel syndrome with diarrhea
4057953	Acute gastric ulcer with perforation
4058821	Primary sclerosing cholangitis
4066036	Gastroduodenal fistula
4069772	Diverticulum of appendix
4135822	Primary biliary cholangitis
4144111	Gastroesophageal reflux disease without esophagitis
4146517	Chronic peptic ulcer with perforation
4150681	Chronic gastric ulcer with perforation
4163865	Acute peptic ulcer without hemorrhage AND without perforation
4164898	Diverticulosis of large intestine without diverticulitis
4173408	Chronic duodenal ulcer with perforation
4174044	Chronic peptic ulcer with hemorrhage
4215489	Calculus of bile duct with chronic cholecystitis without obstruction
4222896	Chronic duodenal ulcer without hemorrhage AND without perforation
4225273	Chronic gastritis
4232181	Chronic duodenal ulcer with hemorrhage
4234788	Irritable bowel syndrome characterized by alternating bowel habit
4260364	Choledochal cyst
4260535	Diverticulitis of large intestine
4261072	Irritable bowel syndrome characterized by constipation
4266809	Diverticular disease
4284982	Calculus of bile duct without obstruction
4288676	Acute and chronic cholecystitis
4294973	Chronic gastric ulcer with hemorrhage and with perforation

4296611	Chronic gastric ulcer without hemorrhage AND without perforation
4300203	Cholelithiasis with obstruction
4316362	Chronic cholecystitis with calculus
36716700	Perforation and abscess of large intestine co-occurrent and due to diverticulitis
37016396	Acute cholecystitis due to biliary calculus
37116441	Diverticulitis of small intestine with perforation and abscess
40482241	Chronic ulcerative pancolitis
40482865	Left sided ulcerative colitis
44783016	Calculus of gallbladder with acute and chronic cholecystitis
45757783	Gastric hemorrhage due to alcoholic gastritis
46269823	Barretts esophagus with dysplasia
46269824	Barretts esophagus with high grade dysplasia
46269825	Barretts esophagus with low grade dysplasia
46269831	Cholangitis due to bile duct calculus with obstruction
46269837	Gastric hemorrhage due to chronic superficial gastritis
46269838	Abscess of intestine co-occurrent and due to chronic ulcerative pancolitis
46269839	Complication due to chronic ulcerative pancolitis
46269840	Fistula of intestine due to chronic ulcerative pancolitis
46269841	Rectal hemorrhage due to chronic ulcerative pancolitis
46269842	Abscess of intestine co-occurrent and due to chronic ulcerative proctitis
46269843	Complication due to chronic ulcerative proctitis
46269844	Fistula of intestine due to chronic ulcerative proctitis
46269847	Rectal hemorrhage due to chronic ulcerative proctitis
46269848	Abscess of intestine co-occurrent and due to chronic ulcerative rectosigmoiditis
46269849	Complication due to chronic ulcerative rectosigmoiditis
46269850	Fistula of large intestine due to chronic ulcerative rectosigmoiditis
46269851	Rectal hemorrhage due to chronic ulcerative rectosigmoiditis
46269874	Complication due to Crohn's disease of large intestine
46269875	Fistula of large intestine due to Crohn's disease
46269876	Intestinal obstruction due to Crohn's disease of large intestine
46269877	Rectal hemorrhage due to Crohn's disease of large intestine
46269878	Abscess of intestine co-occurrent and due to Crohn's disease of small and large intestine
46269879	Complication due to Crohn's disease of small and large intestines
46269880	Fistula of intestine due to Crohn's disease of small and large intestine
46269881	Intestinal obstruction due to Crohn's disease of small and large intestine
46269882	Rectal hemorrhage due to Crohn's disease of small and large intestines
46269883	Abscess of intestine co-occurrent and due to Crohn's disease of small intestine
46269884	Complication due to Crohn's disease of small intestine
46269885	Fistula of small intestine due to Crohn's disease

46269886	Intestinal obstruction due to Crohn's disease of small intestine
46269887	Rectal hemorrhage due to Crohn's disease of small intestine
46269888	Abscess of intestine co-occurrent and due to Crohn's disease
46269889	Complication due to Crohn's disease
46269890	Intestinal obstruction due to Crohn's disease
46269891	Rectal hemorrhage due to Crohn's disease
46269900	Diverticulitis of small and large intestine with perforation
46269907	Intestinal hemorrhage with diverticulosis
46269951	Abscess of intestine co-occurrent and due to ulcerative colitis
46269952	Fistula of intestine due to ulcerative colitis
46270529	Hemorrhage of small intestine with diverticulosis
46273477	Complication due to ulcerative colitis
46273478	Rectal hemorrhage due to ulcerative colitis
46274069	Intestinal obstruction due to chronic ulcerative pancolitis
46274073	Abscess of intestine co-occurrent and due to Crohn's disease of large intestine
46274082	Intestinal obstruction due to ulcerative colitis
