# Supplementary material for: New-onset gastrointestinal disorders in COVID-19 patients 3.5 years post-infection in the inner-city population in the Bronx
Source: Sci Rep. 2024 Dec 30;14:31850. doi: 10.1038/s41598-024-83232-7 (PMC11685902; doi:10.1038/s41598-024-83232-7)
Supplement: Supplementary file 2 — Supplementary Material 2. [file 41598_2024_83232_MOESM2_ESM.pdf]

Supplementary Table 2: Crude and multivariable cox-proportional hazard ratios of developing comorbidities. This data was not matched.

Variable	Crude Hazard Ratio	95% CI	Adjusted Hazard Ratio	95% CI
COVID-19 Positive vs Negative	1.51*	(1.45-1.58)	1.24*	(1.18-1.29)
Age (continuous)	1.02*	(1.02-1.02)	1.01*	(1.01-1.01)
female vs male	1.32*	(1.29-1.35)	1.29*	(1.26-1.32)
Black vs White	0.94*	(0.92-0.97)	1.15*	(1.12-1.19)
Asian vs White	1.05	(0.99-1.12)	1.52*	(1.42-1.61)
Others vs White	0.84*	(0.82-0.87)	0.91*	(0.88-0.94)
Hispanic vs non-Hispanic	1.33*	(1.30-1.35)	1.60*	(1.56-1.64)
Smoker vs Never	2.02*	(1.98-2.06)	1.40*	(1.37-1.43)
Diabetes	2.27*	(2.22-2.32)	1.17*	(1.14-1.19)
Hypertension	2.35*	(2.31-2.40)	1.14*	(1.12-1.18)
COPD	2.51*	(2.37- 2.66)	1.04	(0.98- 1.11)
CKD	2.29*	(2.21-2.37)	1.05*	(1.01-1.09)
Cardiovascular diseases	2.10*	(2.11-2.15)	1.013	(0.97-1.05)
Asthma	1.20*	(1.17-1.24)	1.15*	(1.11-1.18)
Obesity	1.75*	(1.70-1.78)	1.30*	(1.27-1.34)
