# Supplementary material for: New-onset gastrointestinal disorders in COVID-19 patients 3.5 years post-infection in the inner-city population in the Bronx
Source: Sci Rep. 2024 Dec 30;14:31850. doi: 10.1038/s41598-024-83232-7 (PMC11685902; doi:10.1038/s41598-024-83232-7)
Supplement: Supplementary file 3 — Supplementary Material 3. [file 41598_2024_83232_MOESM3_ESM.pdf]

Supplementary Table 3: Incidences of GID for patients infected by different predominant strains with one-year follow-up time. These incidences are not adjusted for covariates.

Strain	Observation period ended	Infected people	Number of New GID after one year of follow-up.
Original (Mar 2020 to May 2020)	May 2021	4157	156 (3.75%)
Alpha (Jan 2021 to Mar 2021)	Mar 2022	3853	151 (3.91%)
Delta (Aug 2021 to Oct 2021)	Oct 2022	1367	34 (2.48%) *
Omicron (Jan 2022 to Feb 2022)	Feb 2023	5567	198 (3.55%)

*** $p < 0.001$, ** $p < 0.01$, * $p < 0.05$ (chi-square test for pairwise comparison)
